# Nutrition status of nulliparous married women (15–24 years) in South Asia: trends, predictors, and program implications

**DOI:** 10.3389/fnut.2024.1445314

**Published:** 2024-11-18

**Authors:** Abhishek Kumar, Vani Sethi, Zivai Murira, Atma Prakash, Anita Shrestha, William Joe

**Affiliations:** ^1^Department of Economics, FLAME University, Pune, Maharashtra, India; ^2^UNICEF Regional Office for South Asia, Kathmandu, Nepal; ^3^Institute of Economic Growth, Delhi, India; ^4^Independent Consultant, Kathmandu, Nepal

**Keywords:** nulliparous women, thinness, severe thinness, overweight, nutrition, South Asia

## Abstract

**Introduction:**

Preconception nutrition, the nutritional status of women before pregnancy, is crucial for maternal and child health. Interventions focusing on preconception nutrition can help break the intergenerational cycle of malnutrition by improving the health and nutritional status of women before pregnancy. This study investigated the recent trends and patterns in the nutritional status of nulliparous adolescents and young women across six countries in South Asia: Bangladesh, India, Maldives, Nepal, Pakistan, and Sri Lanka.

**Methods:**

This study utilized the cross-sectional data from the Demographic and Health Survey (DHS) conducted between 2010 and 2022 for six South Asian countries. A total sample of 20,024 nulliparous married women aged 15–24 years was analyzed to estimate the pooled prevalence for various anthropometric outcomes. Annual changes in the prevalence of the outcome indicators were presented for each country. Predictors of thinness and overweight were analyzed using logistic regression models.

**Results:**

Based on the latest rounds of DHS for respective countries, the pooled weighted prevalence of thinness was 24.4%, overweight was 24.8%, and short height was 11.3%. The prevalence of underweight ranged from 14.6% in Pakistan (DHS 2018) to 25.9% in India (DHS 2021). The least reduction in the prevalence of underweight was observed in India at 2.8% and Nepal at 0.7%. Based on the latest surveys, the mean BMI among women aged 15–24 years was the highest in Maldives (24.1, 95% CI: 23.4, 24.8) and Pakistan (22.9, 95% CI: 22.2, 23.5) and the lowest in India (20.9, 95% CI: 20.9, 21.0) and Nepal (20.8, 95% CI: 20.4, 21.2). The pooled prevalence of thinness and short height was high in rural areas (26.2 and 11.8%), among less educated (28.1 and 14.5%), and bottom 40% wealth quintile groups (29.8 and 15.8%). Compared to young women (20–24 years), adolescent girls were 39% more likely to be underweight (OR: 1.39; 95% CI: 1.25, 1.54).

**Conclusion:**

The findings highlight the need to broaden the scope of policies and programs designed for pregnant and lactating women so that nulliparous married women can be screened frequently for their anthropometric progress. Given the uneven distribution of the burden, it is recommended to implement comprehensive nutrition packages to reach all population subgroups across the regions.

## 1 Introduction

The dietary practices and nutritional status of women in the preconception stage is a critical determinant of maternal and child health outcomes. Substantial evidence shows that children born to women who are undernourished—particularly those who are thin and short—are more likely to be born prematurely and experience low birth weight and intrauterine growth retardation (1–4). High levels of undernutrition among women during the preconception phase are a key factor contributing to the high burden of stunting (5, 6). Women of reproductive age (WRA) and adolescent girls are physiologically susceptible to undernutrition and micronutrient deficiencies due to the increased nutrient requirements for menstruation, pregnancy, and lactation. It is well known that undernutrition among women of reproductive age groups continues to be a public health challenge for most of the countries in South Asia (7–10). Interventions initiated post-conception, such as enhancing maternal diet and micronutrient intake, might enhance maternal nutrition but may have limited influence on newborn health results (11). Given the substantial influence on the well-being of both mothers and children, it is imperative to advocate for and prioritize interventions that focus on enhancing the nutrition of nulliparous women. Nulliparous women (women who have never given birth) represent a unique group within the reproductive age group (15–49 years) with distinct health needs. As the nulliparous women prepare for potential first pregnancies, ensuring adequate intake of key nutrients like folic acid and iron is essential to support healthy maternal and fetal outcomes. Since many young women may not be planning for pregnancy, they are often unaware of their specific nutritional requirements, increasing the risk of deficiencies (4). Additionally, lifestyle factors like poor diet or unhealthy habits further emphasize the importance of targeted interventions to ensure young women are well-prepared for healthy pregnancies in the future (4).

Despite a decrease in the incidence of underweight in WRA from 14.6% in 1975 to 9.7% in 2014, there are still significant burdens across Africa and Asia, with a rate of 24% in South Asia (12). As of 2022, 50% of women in the reproductive age group are anemic in South Asia, while approximately a quarter are thin (7, 13). Even within South Asia, the prevalence of malnutrition varies considerably. While India accounts for the large share of the undernutrition burden, the prevalence of overweight is high across Bangladesh and Maldives (7). Over the last two decades, there has been some progress in reducing the burden of undernutrition. However, the prevalence of overweight and obesity is increasing drastically across the world (7, 9). Consequently, regions including South Asia are now experiencing a double burden of malnutrition (7). Evidence suggests mothers who are obese are likely to suffer from pre-eclampsia and gestational diabetes (1, 11).

While both the high burden of undernutrition and overweight/obesity are of concern, most of the nutrition programs are primarily focused on delivering services to pregnant and lactating women. Recent studies have demonstrated that addressing preconception risk factors, such as undernutrition or obesity, is crucial for reducing adverse health outcomes during pregnancy and in newborns (14–16). For instance, research has shown that poor nutritional status in the preconception stage is associated with higher risks of preterm birth, low birth weight, and intrauterine growth restriction (16). Furthermore, evidence supports the role of maternal preconception health in influencing long-term child development and reducing the intergenerational cycle of malnutrition (15). The focus on addressing preconception nutrition needs is lacking despite an increase in spending on nutrition initiatives. In recent years, various governments and policymakers have recognized the nutrition needs of women in the preconception stage to break the intergenerational cycle of malnutrition.

This study aims to investigate the trends and patterns in the nutritional status of nulliparous women across six countries in South Asia: Bangladesh, India, Maldives, Nepal, Pakistan, and Sri Lanka. The key objectives of this study are to (1) present prevalence of thinness, severe thinness, overweight (including obesity), and short stature among married nulliparous adolescents (15–19 years) and nulliparous young women (20–24 years) for each country in South Asia; (2) estimate the average annual rate of reduction and the annual rate of increase within countries of South Asia using data for two time periods; and (3) identify the predictors of underweight and overweight for adolescents and young women, and to understand the strength of association with the specific predictors across countries over time.

## 2 Materials and methods

The study utilized data from the nationally representative Demographic Health Survey (DHS). DHS provides information on anthropometric outcomes as well as the socio-economic characteristics of the women. For this study, we included six out of eight South Asian countries: Bangladesh, India, Maldives, Nepal, Pakistan, and Sri Lanka. Data for two time periods were considered for each country: Bangladesh (2018 and 2022), India (2016 and 2021), Maldives (2009 and 2017), Nepal (2016 and 2022), and Pakistan (2013 and 2018). Data for Sri Lanka was available only for 2016 limiting the analysis of trends and patterns over time. Data for height and weight were not collected for Afghanistan and Bhutan.

The analysis is based on a total of 20,024 married nulliparous women aged 15–24 years (adolescents—7,188 and young women—12,836), obtained from the latest rounds of DHS for each country (Table 1). Most of the sample was from India (18,388), followed by Bangladesh (503) and Sri Lanka (391).

**Table 1:** Analytical sample and sample size used in the study.

			Out of 15–49 years		After applying exclusion criteria		
		Women 15–49 years	Adolescents (15–19 years)	Young adults (20–24 years)	Adolescents (15–19 years)	Young adults (20–24 years)	Total sample
Bangladesh	DHS 2018	20,127	1,951	3,154	648	400	1,048
	DHS 2022	30,078	2,449	4,851	328	175	503
India	DHS 2016	699,686	124,878	122,955	9,072	13,443	22,515
	DHS 2021	724,115	122,480	118,700	6,520	11,868	18,388
Maldives	DHS 2009	7,131	129	1,381	57	289	346
	DHS 2016	7,699	1,015	1,118	28	225	253
Nepal	DHS 2016	12,862	2,622	2,306	141	145	286
	DHS 2022	14,845	2,777	2,623	128	121	249
Pakistan	DHS 2012	4,112	567	2048	82	133	215
	DHS 2017	15,068	728	2,220	90	150	240
Sri Lanka	DHS 2016	18,302	229	1,439	94	297	391
Overall	Earlier round	743,918	130,147	131,844	10,000	14,410	24,410
Overall	Latest round	810,107	129,678	130,951	7,188	12,836	20,024

Exclusion criteria – women who are pregnant, who have children, whose height and weight were not recorded and who are not married.

### 2.1 Primary outcome indicators of nutritional status

Three indicators of nutritional status were created based on the Asia Pacific cut-off for BMI: thinness (BMI < 18.5 kg/m^2^), severe thinness (BMI < 16 kg/m^2^), and Overweight, including obesity (BMI ≥ 23 kg/m^2^) and short stature (less than 145 cm). BMI was calculated by dividing the person’s weight in kilograms by the square of their height in meters. The variable was then categorized into the different BMI ranges. *Z*-scores for adolescents were calculated using World Health Organization (WHO) reference standards. Growth standards specified by WHO were used to determine the anthropometric outcomes for adolescents by comparing the calculated *z*-score with the median z-score: thinness (BAZ < −2 SD), severe thinness (BAZ < −3 SD) and obesity (BAZ > +1 SD).

Pooled prevalence was estimated after adjusting the sampling weight. A weight was created for each country by dividing the population of women (15–49 years) for each country by the population of women for the SA region. The population was obtained from World Population Prospects for the corresponding survey years (17). Subsequently, this weight was multiplied by the survey sampling weight for each country and used for the analysis of the pooled dataset.

### 2.2 Socio-economic correlates

**Figure 1:**
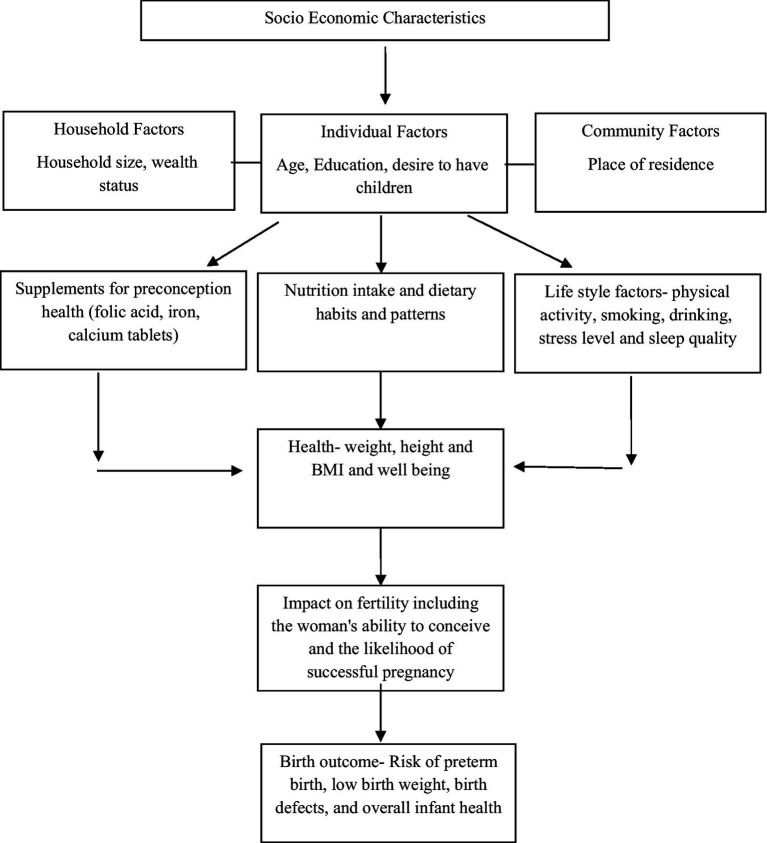
Directed Acyclic Graph (DAG) showing the potential relationship between socio-economic characteristics, anthropometric outcomes and pregnancy and birth outcomes.

Based on the literature, several socio-economic factors associated with undernutrition among women were included in the analysis (Figure 1). Age (15–19 and 20–24 years), place of residence (urban and rural), education (Up to 10 years; more than 10 years), fertility intention (desire for a child/another child sooner or later, that is after 2 years or want no more), and the wealth quintile (bottom 40% and top 60%) provided by DHS based on a list of pre-defined assets were identified as important determinants of malnutrition.

Education was categorized using 10 years as the cut-off, based on both data considerations and the results of our sensitivity analysis. This threshold offered the best balance for minimizing skewness in the distribution of educational attainment across countries while maintaining an adequate sample size for each education category. Similarly, we merged the top three wealth quintiles to ensure sufficient observations within each category and to further reduce skewness in the wealth distribution.

### 2.3 Statistical analysis

Univariate analysis was conducted to examine the prevalence of thinness, severe thinness, and overweight and short stature in South Asia and for each country for both age groups. Bivariate analysis was conducted to estimate the prevalence by age and key socio-economic correlates (Supplementary Table S1). The analysis also included the changes in prevalence over time and across various sub-national regions.

Logistic regression models were applied to determine the association of selected variables with undernutrition among nulliparous women. Models were estimated separately for each undernutrition indicator as the dependent variable. The following econometric specification was used for estimation.


$$\mathrm{logit}\;\pi_{i}=\beta_{1}+\beta_{2}X_{i}+\varepsilon_{i}$$

Where $\pi_{i}$ = Probability ( $Y_{i}=1$ ). $Y_{i}$ is binary and assumes a value of 1 if the woman is thin/overweight and 0 otherwise. Log of odds has been estimated for the model while controlling for several socio-economic and demographic correlates (denoted by X). The control variables which were used in the above equation are age, place of residence, education, fertility intention and wealth quintile. STATA 15 was used for all the statistical analysis. All statistical tests were two-sided and *p* < 0.05 was considered to determine statistical significance.

### 2.4 Annual rate of reduction and annual rate of increase

The Annual Average Rate of Reduction (AARR), which is the average relative percentage decrease per year in prevalence or rate (18), was calculated as follows. Here “n” is the number of years between the time periods denoted by “t” and “n + t,” and $P_{t+n}$ and $P_{t}$ are the prevalence rates of the indicators (thinness/overweight) at time points “t + n” and “t” respectively. The calculation is based on previous and latest rounds of DHS for the respective countries except for Sri Lanka, for which data was available for 2016 only.


AARR=1−Pt+nPt1/n∗100

A positive sign of AARR indicates a reduction or downward trend, while a negative sign indicates an increase or upward trend.

The Annual Average Rate of Increase (AARI), the average relative percentage increase per year in prevalence or rate, was calculated as


AARI=Pt+nPt1n−1∗100

A positive sign of AARI indicates an increase or upward trend, while a negative sign indicates a decrease or a downward trend.

## 3 Results

Based on the latest rounds of DHS for respective countries, the pooled weighted prevalence of thinness was 24.4%, overweight was 24.8%, and short height was 11.3%. In South Asia, underweight prevalence has decreased: Bangladesh from 20.4 to 18.2% (2018–2022), India from 29.9 to 25.9% (2016–2021), Maldives from 26.4 to 15.9% (2009–2017), Pakistan from 17.6 to 14.6% (2013–2018), Nepal from 23.3 to 22.4% (2016–2022), and Sri Lanka at 18.4% (2016). Conversely, overweight prevalence increased in most countries: Bangladesh from 29.7 to 32.4%, India from 17.1 to 22.2%, Maldives from 35.4 to 55.1%, and Pakistan from 27.2 to 45.3%, while Nepal decreased slightly from 19.5 to 17.9%, and Sri Lanka reported 43.3% (2016).

### 3.1 Change in nutritional status

Substantial variation was observed in the annual rate of reduction in the prevalence of underweight and the annual rate of increase in the prevalence of overweight across countries among married nulliparous adolescents and married young women (15–24 years) (Table 2). Maldives had the highest AARR in underweight prevalence (6.1%) while Nepal showed a minimal reduction of 0.7% from 23.3% in 2016 and 22.4% in 2022. The AARR in underweight prevalence was 2.9% in Bangladesh, 2.8% in India, and 3.7% in Pakistan. The AARI in the prevalence of overweight was highest in Pakistan (10.7%) followed by Maldives (5.7%), India (5.4%) and Bangladesh (2.2%). Nepal is the only country experiencing a reduction in overweight prevalence with AARR of 1.4%. However, the prevalence of thinness has increased among young women (20–24 years) in the country.

**Table 2:** Annual rate of reduction/increase in prevalence of thinness (BMI < 18.5 kg/m^2^) and overweight (BMI ≥ 23 kg/m^2^) and height less than 145 cm among married nulliparous adolescents and young women, South Asia.

			Overall			Adolescents (15–19 years)				Young women (20–24 years)			
	Round	*N*	<18.5 kg/m^2^ (%)	≥ 23 kg/m^2^ (%)	Less than 145 cm (%)	*N*	<18.5 kg/m^2^ (%)	≥ 23 kg/m^2^ (%)	Less than 145 cm (%)	*N*	<18.5 kg/m^2^ (%)	≥ 23 kg/m^2^ (%)	Less than 145 cm (%)
Bangladesh	DHS 2018	1,048	20.4	29.7	12.7	648	24.7	21.7	9.7	400	13.3	43.2	17.8
	DHS 2022	503	18.2	32.4	5.7	328.0	20.8	28.9	5.5	175.0	13.1	39.0	6.1
	Change		(2.9)	2.2	(18.3)		(4.2)	7.4	(13.3)		(0.3)	(2.5)	(23.6)
India	DHS 2016	22,515	29.9	17.1	11.9	9,072	35	10.9	13.1	13,443	26.3	21.4	11.1
	DHS 2021	18,388	25.9	22.2	12.3	6,520	31.6	14.4	14.9	11,868	22.4	26.8	10.7
	Change		(2.8)	5.4	0.7		(2.0)	5.7	2.6		(3.2)	4.6	(0.7)
Maldives	DHS 2009	346	26.4	35.4	6.6	57	27.2	31.3	5.6	289	26.2	36.1	6.8
	DHS 2017	253	15.9	55.1	2.5	28	22.4	66.4	7.6	225	15.3	54.1	2.1
	Change		(6.1)	5.7	(11.4)		(2.4)	9.9	3.9		(6.5)	5.2	(13.7)
Nepal	DHS 2016	286	23.3	19.5	10.9	141	30.9	9.4	13.4	145	16.3	28.9	8.7
	DHS 2022	249	22.4	17.9	9.4	128	23.9	15.3	6.5	121	21	20.4	12.1
	Change		(0.7)	(1.4)	(2.4)		(4.2)	8.5	(11.4)		4.3	5.6	5.7
Pakistan	DHS 2013	215	17.6	27.2	8.1	82	18	16	13.7	133	17.3	34.3	4.6
	DHS 2018	240	14.6	45.3	6.6	90	19.6	41.3	11.8	150	12.2	47.1	4.2
	Change		(3.7)	10.7	(4.0)		1.7	20.9	(2.9)		(6.7)	6.5	(1.8)
Sri Lanka	DHS 2016	391	18.4	43.3	5.5	94	22.9	37.5	6.4	297	17	45.2	5.2
Pooled	Earlier round	24,410	27.6	19.7	11.8	10,000	31.8	13.6	12.4	14,410	24.2	24.7	11.4
	Latest round	20,024	24.4	24.8	11.3	7,188	29.5	17.9	13.4	12,836	21.1	29.2	9.9

Pooled estimates for the earlier round do not include Sri Lanka as the survey was not conducted. Figures in parenthesis are AARR. Figures without parenthesis are AARI.

The AARR in the prevalence of short stature in the Bangladesh and Maldives was 18.3 and 11.4%, respectively. In, India, the percentage of short women has increased slightly in the latest DHS rounds. The AARR for short stature in young women (20–24 years) was highest in Bangladesh (23.6%).

**Figure 2:**
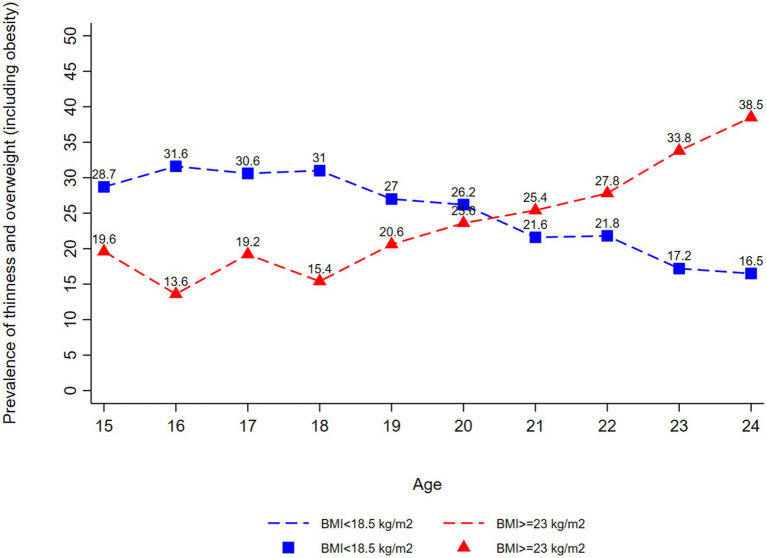
Thinness (BMI < 18.5 kg/m^2^) and overweight or obesity (BMI ≥ 23 kg/m^2^) among married nulliparous adolescents (15–19 years) and married nulliparous young women (20–24 years) by Age, South Asia based on the latest rounds for respective countries.

According to the latest round of surveys, India had the highest prevalence of underweight at 25.9%, followed by Nepal (22.4%) (Supplementary Table S2). Overweight prevalence is very high in Maldives (55.1%), Pakistan (45.3%), and Sri Lanka (43.3%) (Supplementary Table S3). India has the highest prevalence of short stature at 12.3% (Supplementary Table S4). The study noted a decline in the prevalence of thinness from age 20 years while the prevalence of obesity increased with the age of the women (Figure 2).

The prevalence of anemia was assessed for three countries: India, Maldives, and Nepal (Supplementary Table S6). Based on the latest surveys, the anemia prevalence rate was high in India and Maldives at approximately 59%. The rate increased from 52.8% in 2016 to 58.3% in 2021 in India while it remained nearly static in Nepal (approximately 38%).

### 3.2 Mean BMI and nutritional status based on BMI-for-*z* score

Based on the latest round of surveys, the mean BMI for women aged 15–24 years varied across South Asian countries, with the highest values observed in Maldives (24.1), followed closely by Pakistan (22.9) and Sri Lanka (22.8), indicating relatively better nutritional status in these regions (Supplementary Figure S1). Conversely, the lowest mean BMI was recorded in Nepal (20.8) and India (20.9), suggesting a greater prevalence of undernutrition among young women in these countries. Furthermore, mean BMI was observed to be higher for young women as compared to the adolescents (Supplementary Figures S2, S3).

The prevalence of thinness, assessed through the BMI-for-*z* score, revealed a concerning situation: Pakistan exhibited the highest prevalence of thinness at 10.6%, followed by India at 7.2%, Maldives at 6.7%, and Sri Lanka at 7.9% (Table 3). This data highlights the nutritional challenges faced by young women in these countries. Notably, Maldives also reported a striking prevalence of overweight at 61.5% based on the BMI-for-*z* score, indicating a significant shift toward higher weight categories among its young female population.

**Table 3:** Prevalence of thinness (BAZ < −2SD), severe thinness (BAZ < −3SD) and overweight or obesity (BMI + 1SD) among married nulliparous adolescents (15–19 years), South Asia.

			*z*-score of BMI for age		
	Round	*N*	<−2SD (%)	<−3SD (%)	>+1SD (%)
Bangladesh	DHS 2018	648	5.3	0.4	12.6
	DHS 2022	328	3.1	0.42	17.3
India	DHS 2016	9,072	7.1	1.1	5.3
	DHS 2021	6,520	7.2	1	7.1
Maldives	DHS 2009	57	13	4.9	18.1
	DHS 2017	28	6.7	2.2	61.5
Nepal	DHS 2016	141	3.5	0	3.5
	DHS 2022	128	1.6	0	5.9
Pakistan	DHS 2013	82	3.1	1.3	7.5
	DHS 2018	90	10.6	0	27.7
Sri Lanka	DHS 2016	94	7.9	0	27.5
Pooled	Earlier	10,000	6.4	0.9	7.1
	Latest	7,188	6.8	0.8	9.7

### 3.3 Socio-economic distribution

On average, 24.4% of women resided in urban areas. Approximately 50% of them had more than 10 years of education. A high proportion of women (89.8%) desired children soon. In the pooled sample based on wealth, 57.1% belonged to the top 60% wealth quintile group while 42.9% belonged to the bottom 40% group. More than 60% of women resided in households with more than four members. Overall, 61.2% of women were 20–24 years old, and 38.8% were in the 15–19 years age group. The country wise distribution of participants by socio-economic characteristics is shown in Supplementary Table S1.

The study observed a high prevalence of thinness and short height in rural areas (26.2 and 11.8%), among less educated (28.1 and 14.5%), and the bottom 40% wealth quintile groups (29.8 and 15.8%). The prevalence of thinness was high in household sizes greater than 4 (25.2%) and among 15–19 years (29.5%), while the prevalence of overweight was high in small households (27.2%) and among 20–24 years age group women (29.2%) (Table 4). The patterns are consistent across all countries (Supplementary Tables S2, S3).

**Table 4:** Prevalence of thinness (BMI < 18.5 kg/m^2^) and overweight (BMI ≥ 23 kg/m^2^) and height less than 145 cm among married nulliparous adolescents and young women, South Asia based on the latest round of DHS for respective countries.

	Sample		BMI < 18.5 kg/m^2^	BMI ≥ 23 kg/m^2^	Height < 145 cm
	*N*	%	%	%	%
Sector					
Rural	16,366	75.6	26.2	22.5	11.8
Urban	3,658	24.4	18.6	31.9	9.8
Education respondent					
Up to 10 years	9,723	48.3	28.1	20.6	14.5
More than 10 years	10,301	51.7	20.9	28.7	8.3
Desire for children					
Want soon	17,631	89.8	23.9	25.5	11.1
Want no more/Others	2,393	10.2	28.5	18.9	12.7
Wealth					
Bottom 40%	9,324	42.9	29.8	17.2	15.8
Top 60%	10,700	57.1	20.3	30.5	7.9
Household size					
≤4	7,812	38.4	23	27.2	12
>4	12,212	61.6	25.2	23.3	10.8
Woman age					
15–19 years	7,188	38.8	29.5	17.9	13.4
20–24 years	12,836	61.2	21.1	29.2	9.9
Total	20,024	100	24.4	24.8	11.3

Table 5 presents the association between underweight and the socio-economic characteristics among married nulliparous adolescents and young women aged 15–24 years across the countries, based on the recent rounds of DHS. Compared to young women (20–24 years), adolescent girls were 39% more likely to be underweight (OR: 1.39; 95% CI: 1.25, 1.54). Women residing in urban areas (OR:0.78: 95% CI:0.68, 0.89) and those with more than 10 years of education (OR:0.84: 95% CI:0.76, 0.93) were significantly less likely to be underweight than those in rural areas and with a low education. Women belonging to the top 60% were less likely to be underweight (OR:0.72: 95% CI:0.65, 0.80).

**Table 5:** Adjusted odds ratios of underweight among married nulliparous adolescents (15–19 years) and young women (20–24 years).

	Bangladesh DHS 2022	India DHS 2021	Maldives DHS 2017	Nepal DHS 2022	Pakistan DHS 2018	Sri Lanka DHS 2016	Pooled
							Latest rounds
Sector							
Rural	1	1	1	1	1	1	1
Urban	1.37	0.77***	4.61*	1.39	0.68	0.9	0.78***
	[0.78, 2.43]	[0.67, 0.88]	[1.14, 18.74]	[0.71, 2.71]	[0.26, 1.76]	[0.35, 2.29]	[0.68, 0.89]
Education respondent							
Up to 10 years	1	1	1	1	1	1	1
More than 10 years	0.58	0.79***	1	0.97	1.05	1.14	0.84***
	[0.32, 1.06]	[0.72, 0.87]	[1.00, 1.00]	[0.49, 1.89]	[0.29, 3.78]	[0.31, 4.13]	[0.76, 0.93]
Desire for children							
Want soon	1	1	1	1	1	1	1
Want no more/Others	0.61	1.23**	1.86	5.25	0.06*	1.03	1.21**
	[0.27, 1.39]	[1.08, 1.39]	[0.28, 12.53]	[0.61, 45.39]	[0.01, 0.58]	[0.45, 2.35]	[1.07, 1.38]
Wealth							
Bottom 40%	1	1	1	1	1	1	1
Top 60%	0.62	0.76***	0.51	0.88	0.51	0.67	0.72***
	[0.35, 1.09]	[0.69, 0.83]	[0.11, 2.41]	[0.45, 1.74]	[0.16, 1.63]	[0.39, 1.15]	[0.65, 0.80]
Household size							
≤4	1	1	1	1	1	1	1
>4	1.16	1.07	3.58	1.18	2.96	1.05	1.09
	[0.68, 1.96]	[0.97, 1.17]	[0.88, 14.55]	[0.60, 2.33]	[0.93, 9.43]	[0.61, 1.81]	[0.99, 1.21]
Woman age							
20–24 years	1	1	1	1	1	1	1
15–19 years	1.45	1.40***	1.97	1.13	1.82	1.39	1.39***
	[0.78, 2.71]	[1.28, 1.54]	[0.45, 8.59]	[0.56, 2.27]	[0.67, 4.93]	[0.76, 2.54]	[1.25, 1.54]
Constant	0.25***	0.39***	0.04***	0.21***	0.09***	0.22*	0.36***
	[0.12, 0.50]	[0.36, 0.43]	[0.01, 0.16]	[0.08, 0.52]	[0.03, 0.28]	[0.06, 0.89]	[0.32, 0.40]
*N*	503	18,388	242	249	206	391	19,990

Note: Levels of significance: * for *p* < 0.05, ** for *p* < 0.01, and *** for *p* < 0.001.

Table 6 presents adjusted odds ratios of overweight and the various socio-economic and demographic factors for women among married nulliparous adolescents (15–19 years) and young women (20–24 years). The results showed significantly higher chances of being overweight for the top 60% wealth quintile groups compared to the bottom 40% group (OR: 1.79; 95% CI: 1.56, 2.09). The adolescent girls aged 15–19 years had significantly lower chances of being overweight compared to young women aged 20–24 years (OR: 0.61; 95% CI: 0.54, 0.70). The chances of being overweight were significantly lesser for those residing in households with 4 or more members (OR: 0.83; 95% CI: 0.74;0.94).

**Table 6:** Adjusted odds ratios of overweight among married nulliparous adolescents (15–19 years) and young women (20–24 years).

	Bangladesh DHS 2022	India DHS 2021	Maldives DHS 2017	Nepal DHS 2022	Pakistan DHS 2018	Sri Lanka DHS 2016	Pooled
							Latest rounds
Sector							
Rural	1	1	1	1	1	1	1
Urban	1.07	1.35***	0.52	1.23	0.49	0.79	1.23**
	[0.65, 1.75]	[1.18, 1.54]	[0.24, 1.12]	[0.57, 2.63]	[0.21, 1.17]	[0.39, 1.62]	[1.06, 1.41]
Education respondent							
Up to 10 years	1	1	1	1	1	1	1
More than 10 years	1.45	1.30***	1.88	0.68	0.86	0.62	1.12
	[0.92, 2.29]	[1.17, 1.45]	[0.55, 6.45]	[0.31, 1.48]	[0.33, 2.24]	[0.22, 1.75]	[0.98, 1.27]
Desire for children							
Want soon	1	1	1	1	1	1	1
Want no more/Others	0.71	0.75***	0.37	1	2.09	0.56	0.72***
	[0.29, 1.73]	[0.64, 0.88]	[0.12, 1.18]	[1.00, 1.00]	[0.38, 11.43]	[0.27, 1.16]	[0.61, 0.86]
Wealth							
Bottom 40%	1	1	1	1	1	1	1
Top 60%	1.72*	1.63***	1.04	2.44*	3.19*	1.21	1.79***
	[1.05, 2.84]	[1.45, 1.83]	[0.52, 2.08]	[1.05, 5.68]	[1.27, 7.97]	[0.78, 1.87]	[1.56, 2.06]
Household size							
≤4	1	1	1	1	1	1	1
>4	0.79	0.81***	0.53	0.86	0.63	0.95	0.83**
	[0.51, 1.22]	[0.73, 0.89]	[0.22, 1.28]	[0.40, 1.88]	[0.26, 1.54]	[0.62, 1.46]	[0.74, 0.94]
Woman age							
20–24 years	1	1	1	1	1	1	1
15–19 years	0.76	0.56***	1.55	0.72	0.82	0.74	0.61***
	[0.47,1.23]	[0.50,0.62]	[0.50,4.88]	[0.34,1.53]	[0.38,1.75]	[0.45,1.24]	[0.54,0.70]
Constant	0.38**	0.24***	1.53	0.16***	0.81	1.29	0.28***
	[0.19, 0.74]	[0.21, 0.27]	[0.40, 5.87]	[0.06, 0.47]	[0.29, 2.24]	[0.44, 3.81]	[0.24, 0.33]
*N*	503	18,388	253	244	206	391	19,990

Note: Levels of significance: * for *p* < 0.05, ** for *p* < 0.01, and *** for *p* < 0.001.

Table 7 shows the adjusted odds ratios of short stature (height less than 145 cm) and socio-economic and demographic factors among married nulliparous adolescents (15–19 years) and young women (20–24 years). Adolescent girls and young women belonging to the top 60% wealth quintile were less likely to be of short stature than those belonging to the bottom 40% wealth quintile (OR: 0.52; 95% CI: 0.45, 0.60).

**Table 7:** Adjusted odds ratios of short stature (height less than 145 cm) among married nulliparous adolescents (15–19 years) and young women (20–24 years).

	Bangladesh DHS 2022	India DHS 2021	Maldives DHS 2017	Nepal DHS 2022	Pakistan DHS 2018	Sri Lanka DHS 2016	Pooled
							Latest rounds
Sector							
Rural	1	1	1	1	1	1	1
Urban	0.44	1.17	1	1.7	2.99	0.69	1.14
	[0.18, 1.10]	[0.97, 1.41]	[1.00, 1.00]	[0.65, 4.41]	[0.56, 15.97]	[0.16, 2.95]	[0.93, 1.39]
Education respondent							
Up to 10 years	1	1	1	1	1	1	1
More than 10 years	0.73	0.66***	1	0.74	0.17	0.28	0.68***
	[0.33, 1.65]	[0.58, 0.75]	[1.00, 1.00]	[0.23, 2.32]	[0.01, 1.90]	[0.07, 1.07]	[0.59, 0.79]
Desire for children							
Want soon	1	1	1	1	1	1	1
Want no more/Others	0.83	1.1	1	7.14	0.41	1	1.12
	[0.23, 2.98]	[0.92, 1.32]	[1.00, 1.00]	[0.70, 72.98]	[0.04, 4.24]	[0.21, 4.76]	[0.93, 1.34]
Wealth							
Bottom 40%	1	1	1	1	1	1	1
Top 60%	0.67	0.52***	0.41	0.54	0.84	0.86	0.52***
	[0.31, 1.46]	[0.45, 0.59]	[0.10, 1.69]	[0.18, 1.59]	[0.17, 4.13]	[0.34, 2.18]	[0.45, 0.60]
Household size							
≤4	1	1	1	1	1	1	1
>4	1.14	0.85*	0.77	2.45	1.82	0.89	0.89
	[0.52, 2.54]	[0.75, 0.96]	[0.18, 3.26]	[0.85, 7.04]	[0.27, 12.03]	[0.36, 2.18]	[0.78, 1.01]
Woman age							
20–24 years	1	1	1	1	1	1	1
15–19 years	0.76	1.22**	2.27	0.39	2.34	1.12	1.19*
	[0.35, 1.66]	[1.08, 1.38]	[0.45, 11.47]	[0.13, 1.17]	[0.61, 9.02]	[0.39, 3.24]	[1.04, 1.36]
Constant	0.12***	0.23***	0.09***	0.09***	0.03**	0.22*	0.20***
	[0.05, 0.26]	[0.20, 0.26]	[0.02, 0.31]	[0.03, 0.30]	[0.00, 0.33]	[0.05, 0.98]	[0.17, 0.23]
*N*	503	18,388	190	249	206	391	19,990

Note: Levels of significance: * for *p* < 0.05, ** for *p* < 0.01, and *** for *p* < 0.001.

## 4 Discussion

The nutrition status of nulliparous married women remains an under-researched area despite having significant implications for birth outcomes as well as maternal health and wellbeing. This is one of the first comprehensive studies presenting the trends and patterns in thinness, overweight and short stature among nulliparous married women in six South Asian countries. The pooled prevalence of underweight was higher than the pooled prevalence of overweight. The analysis revealed a huge variation in the prevalence of thinness and overweight across the countries. The prevalence of thinness was high in India while the prevalence of overweight was more than 40% across Maldives, Pakistan, and Sri Lanka.

The findings showed place of residence, education, wealth, and age were important determinants of malnutrition. These results were in line with the findings of a recent critical review on malnutrition in South Asia which revealed poverty and food insecurity are the root causes of malnutrition (19). This study highlights a strong gradient concerning wealth which indicates poverty, by affecting the purchasing power of the households, could lead to food insecurity or consumption of a diet with low nutritional value. This linkage can also be established through the relationship between household size and the prevalence of thinness. The finding is consistent with a Turkish study which revealed the highest prevalence of thinness among adolescent students (aged 14–18 years) with the lowest household income and the largest household size (20).

India shares a large burden of malnutrition due to its large population. As per the estimates, 2.3 million married nulliparous women were thin (Supplementary Table S5). Notably, women in rural areas in India were likely to be underweight. The poor nutritional status can be attributed to gender inequalities in nutrition from an early age which has also been documented in a previous study (21).

The study noted a high prevalence of obesity with increasing urbanization. Previous research has demonstrated that urban populations were likely to be overweight/obese due to a sedentary lifestyle, intake of energy-dense foods, and lower engagement in physical activity (22, 23). Although there has been a consistent reduction in the prevalence of underweight, the rate of overweight has increased across countries, such as Maldives and Pakistan. This study found mean BMI increased by 11% in Maldives between 2009 and 2016 (Supplementary Figure S1). The results corroborated with previous studies assessing the trends in the prevalence of double burden of malnutrition which reported mean BMI was increasing across countries (7, 24, 25).

The annual rate of reduction in the prevalence of thinness was lowest in Nepal and India. In addition, the prevalence of severe thinness in India stagnated while the prevalence of anemia increased (Supplementary Tables S6, S7). A previous study noted a similar finding that the prevalence of anemia remained the same despite improvements in overall nutritional status (26). The author noted that AARI in case of India was at 2 for 15–19 years and 3.2% for 20–24 years, indicating the reduction rate has not changed despite policy impetus. The current study observed a slight decline in the prevalence of thinness in Nepal. On the contrary, previous studies based on earlier DHS survey rounds have reported Nepal’s undernutrition rate has rapidly decreased (10, 27).

## 5 Limitations of the study

This study has several limitations. First, the study was based on cross-sectional data and therefore causality could not be inferred from the analysis. Second, the data does not consider seasonality which might affect the measurement of weight. Third, the study only included socio-economic and demographic characteristics. The genetic factors such as maternal and paternal anthropometric outcomes and dietary behavior, which are important determinants of nutrition, were not considered due to data unavailability. Another outcome that the study could not capture was anemia for all countries in South Asia. Only two of the countries (Nepal and India) had information available on hemoglobin. Lastly, there was significant heterogeneity in the time of data collection for each country. For instance, the last DHS was conducted in 2022 in Nepal and Sri Lanka and in 2021 in India. Therefore, the pooled analysis might not reflect the present nutrition status of women. The small sample size for most of the countries might affect the reliability of the estimates.

## 6 Future directions and conclusions

The study found an increase in overweight prevalence and a decline in underweight prevalence across the six South Asian countries, except Nepal. Despite the improvement in undernutrition status, the steady rise in the rates of overweight poses a serious challenge, particularly for Maldives and Pakistan. Socio-economic factors such as age, residence, education, wealth, and household size were significant determinants of nutritional status and should be considered in policy interventions by developing a multisectoral strategy. Similarly, camps within school premises could be organized for adolescent girls to monitor the progress in nutritional outcomes over time. Additionally, system strengthening efforts are required to expand the coverage of nutrition counseling services to assist adolescent girls, women, and their families in making decisions and taking action to improve nutritional outputs. Finally, the governments across these nations need to identify high-burden regions and prepare action plans tailored to their needs based on socio-economic characteristics. Future research should include nulliparous married women in nutrition monitoring and analyze dietary behavior to inform policies. These findings would highlight the need for evidence-based interventions to ensure equitable access to adequate nutrition and a healthier future for nulliparous women in South Asia.

## Data Availability

Publicly available datasets were analyzed in this study. This data can be found at: https://dhsprogram.com/data/available-datasets.cfm.
