# Supplementary material for: Nutrition status of nulliparous married women (15–24 years) in South Asia: trends, predictors, and program implications
Source: Front Nutr. 2024 Nov 18;11:1445314. doi: 10.3389/fnut.2024.1445314 (PMC11608968; doi:10.3389/fnut.2024.1445314)
Supplement: Supplementary file 1 [file Data_Sheet_1.docx]

**Supplementary Material**

Figure S1: Mean BMI among married nulliparous adolescents and young women, South Asia


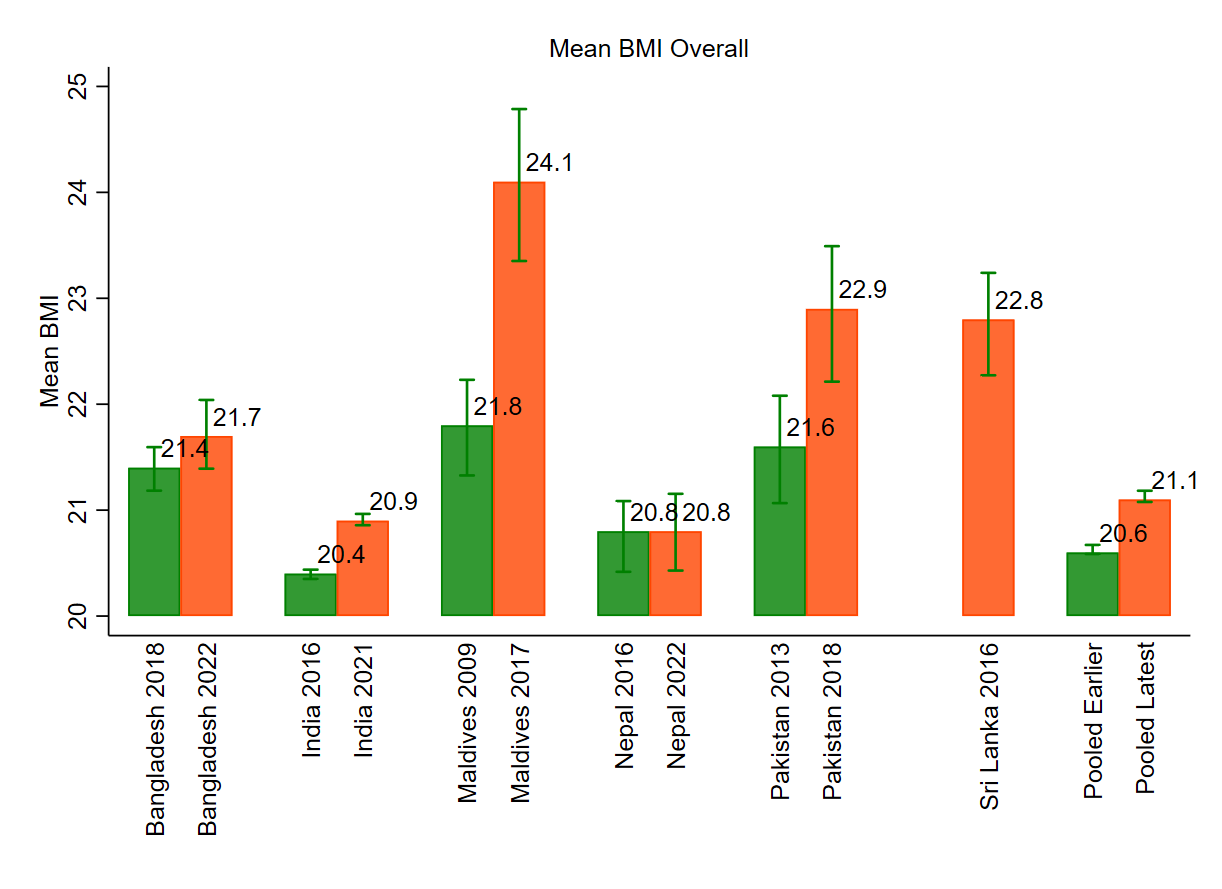


Figure S2: Mean BMI among married nulliparous adolescents (15-19 years), South Asia


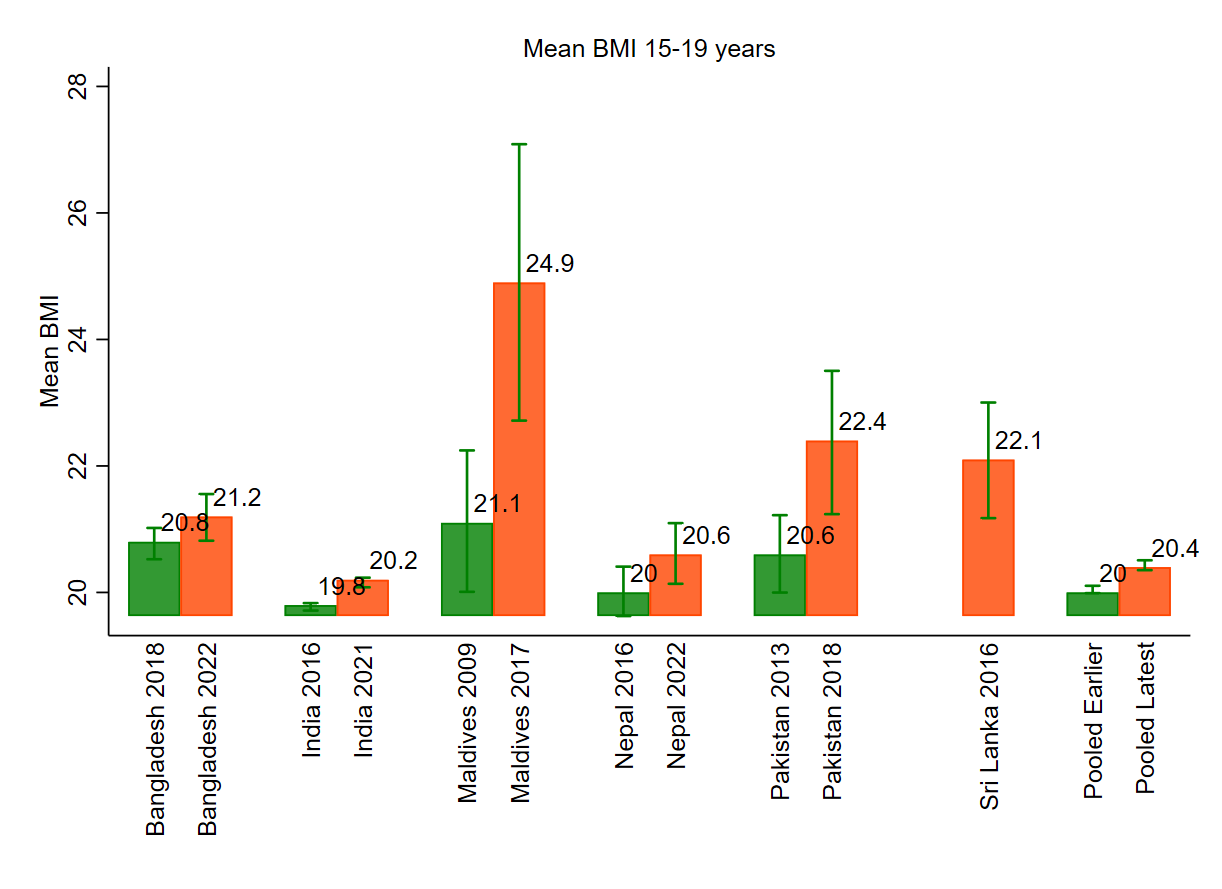


Figure S3: Mean BMI among married nulliparous young women (20-24 years), South Asia


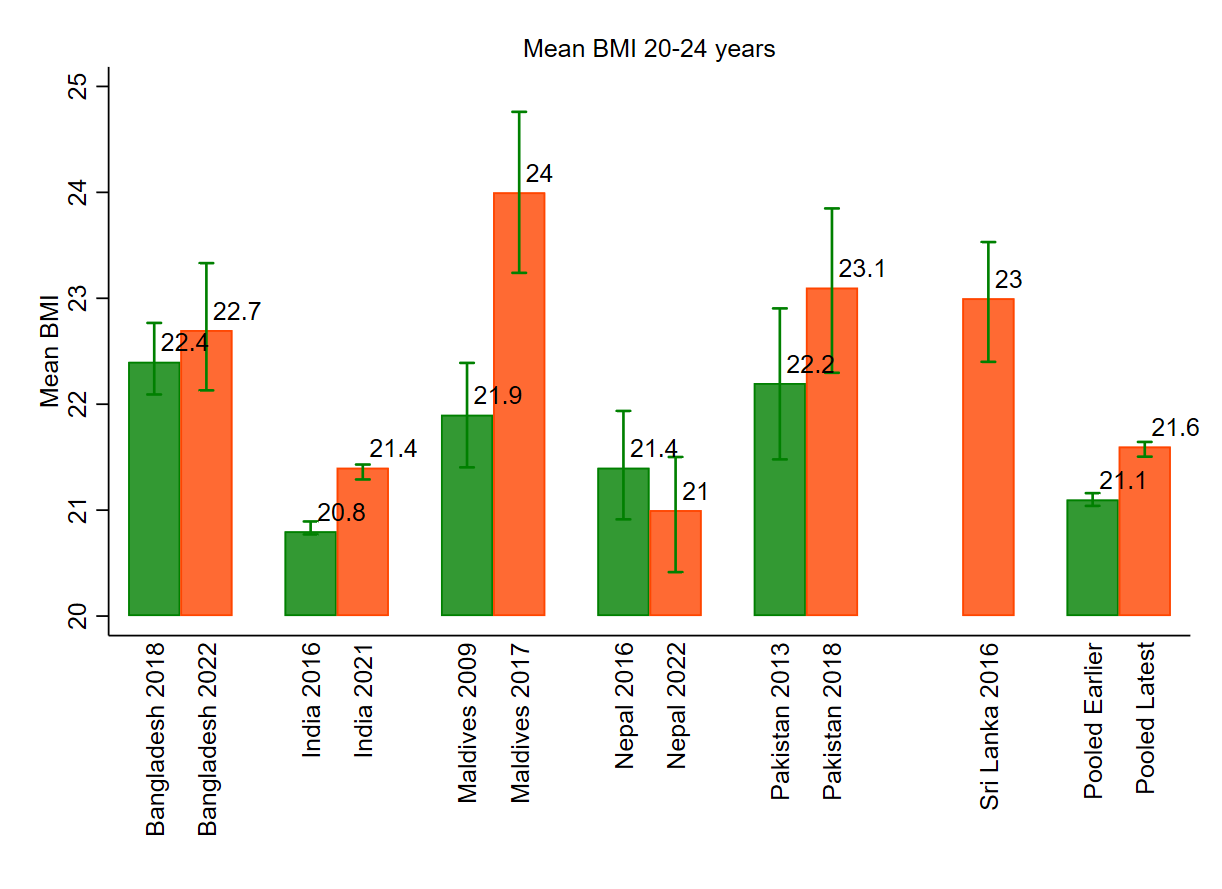


Table S1: Background characteristics, married nulliparous adolescents and young women, South Asia

|  | Bangladesh DHS 2022 | | India DHS 2021 | | Maldives DHS 2017 | | Nepal DHS 2022 | | Pakistan DHS 2018 | | Sri Lanka DHS 2016 | |
| --- | --- | --- | --- | --- | --- | --- | --- | --- | --- | --- | --- | --- |
|  | N | % | N | % | N | % | N | % | N | % | N | % |
| Sector |  |  |  |  |  |  |  |  |  |  |  |  |
| Rural | 330 | 71.2 | 15233 | 77.4 | 214 | 52 | 110 | 32.9 | 130 | 65.6 | 349 | 89.7 |
| Urban | 173 | 28.8 | 3155 | 22.6 | 39 | 48 | 139 | 67.1 | 110 | 34.4 | 42 | 10.3 |
| Education Respondent |  |  |  |  |  |  |  |  |  |  |  |  |
| Up to 10 years | 279 | 56.4 | 9106 | 46.2 | 11 | 3 | 134 | 53.4 | 172 | 68.9 | 21 | 4.5 |
| More than 10 years | 224 | 43.6 | 9282 | 53.8 | 242 | 97 | 115 | 46.6 | 68 | 31.1 | 370 | 95.5 |
| Desire for children |  |  |  |  |  |  |  |  |  |  |  |  |
| Want soon | 461 | 91.9 | 16116 | 88.9 | 238 | 95.6 | 244 | 98.4 | 222 | 96.3 | 350 | 88.8 |
| Want no more/Others | 42 | 8.1 | 2272 | 11.1 | 15 | 4.4 | 5 | 1.6 | 18 | 3.7 | 41 | 11.2 |
| Wealth |  |  |  |  |  |  |  |  |  |  |  |  |
| Bottom 40% | 157 | 31.4 | 8649 | 44.1 | 116 | 31.5 | 122 | 40.5 | 101 | 41.2 | 179 | 43.7 |
| Top 60% | 346 | 68.6 | 9739 | 55.9 | 137 | 68.5 | 127 | 59.5 | 139 | 58.8 | 212 | 56.3 |
| Household size |  |  |  |  |  |  |  |  |  |  |  |  |
| <=4 | 202 | 42.1 | 7183 | 39.1 | 56 | 17.8 | 111 | 45.3 | 50 | 24.6 | 210 | 54.4 |
| >4 | 301 | 57.9 | 11205 | 60.9 | 197 | 82.2 | 138 | 54.7 | 190 | 75.4 | 181 | 45.6 |
| Woman age |  |  |  |  |  |  |  |  |  |  |  |  |
| 20-24 years | 175 | 34.2 | 11868 | 62.7 | 225 | 92.3 | 121 | 51.3 | 150 | 68.6 | 297 | 76.2 |
| 15-19 years | 328 | 65.8 | 6520 | 37.3 | 28 | 7.7 | 128 | 48.7 | 90 | 31.4 | 94 | 23.8 |
| Overall | 503 | 100 | 18388 | 100 | 253 | 100 | 249 | 100 | 240 | 100 | 391 | 100 |

Table S2: Prevalence of thinness (BMI < 18.5 kg/m2) among married nulliparous adolescents and young women, South Asia

|  | Bangladesh DHS 2022 | | India DHS 2021 | | Maldives DHS 2017 | | Nepal DHS 2022 | | Pakistan DHS 2018 | | Sri Lanka DHS 2016 | |
| --- | --- | --- | --- | --- | --- | --- | --- | --- | --- | --- | --- | --- |
|  | N | % | N | % | N | % | N | % | N | % | N | % |
| Sector |  |  |  |  |  |  |  |  |  |  |  |  |
| Rural | 330 | 17.7 | 15233 | 27.7 | 214 | 8.5 | 110 | 18.9 | 130 | 17 | 349 | 18.8 |
| Urban | 173 | 19.2 | 3155 | 19.5 | 39 | 23.8 | 139 | 24.1 | 110 | 10 | 42 | 15.3 |
| Education Respondent | | |  |  |  |  |  |  |  |  |  |  |
| upto 10 years | 279 | 22.5 | 9106 | 30.3 | 11 | 0 | 134 | 23.6 | 172 | 16.6 | 21 | 17.6 |
| More than 10 years | 224 | 12.6 | 9282 | 22.1 | 242 | 16.3 | 115 | 21.1 | 68 | 10.1 | 370 | 18.5 |
| Desire for children | |  |  |  |  |  |  |  |  |  |  |  |
| Want soon | 461 | 18.5 | 16116 | 25.3 | 238 | 15.4 | 244 | 21.8 | 222 | 15.1 | 350 | 18.2 |
| Want no more/Others | 42 | 13.9 | 2272 | 30.2 | 15 | 25.2 | 5 | 60.4 | 18 | 1.4 | 41 | 19.9 |
| Wealth |  |  |  |  |  |  |  |  |  |  |  |  |
| Bottom 40% | 157 | 23.6 | 8649 | 31.2 | 116 | 11.9 | 122 | 23.5 | 101 | 19.6 | 179 | 22.1 |
| Top 60% | 346 | 15.7 | 9739 | 21.7 | 137 | 17.7 | 127 | 21.7 | 139 | 11 | 212 | 15.6 |
| Household size | |  |  |  |  |  |  |  |  |  |  |  |
| <=4 | 202 | 17.4 | 7183 | 24.6 | 56 | 5.5 | 111 | 20.9 | 50 | 6.8 | 210 | 18.5 |
| >4 | 301 | 18.7 | 11205 | 26.7 | 197 | 18.1 | 138 | 23.7 | 190 | 17.1 | 181 | 18.3 |
| Woman age | |  |  |  |  |  |  |  |  |  |  |  |
| 20-24 years | 175 | 13.1 | 11868 | 22.4 | 225 | 15.3 | 121 | 21 | 150 | 12.2 | 297 | 17 |
| 15-19 years | 328 | 20.8 | 6520 | 31.6 | 28 | 22.4 | 128 | 23.9 | 90 | 19.6 | 94 | 22.8 |
| Overall | 503 | 18.2 | 18388 | 25.9 | 253 | 15.9 | 249 | 22.4 | 240 | 14.6 | 391 | 18.4 |

Table S3: Prevalence of overweight (BMI >= 23 kg/m2) among married nulliparous adolescents and young women, South Asia

|  | Bangladesh DHS 2022 | | India DHS 2021 | | Maldives DHS 2017 | | Nepal DHS 2022 | | Pakistan DHS 2018 | | Sri Lanka DHS 2016 | |
| --- | --- | --- | --- | --- | --- | --- | --- | --- | --- | --- | --- | --- |
|  | N | % | N | % | N | % | N | % | N | % | N | % |
| Sector |  |  |  |  |  |  |  |  |  |  |  |  |
| Rural | 330 | 30.9 | 15233 | 19.7 | 214 | 62.7 | 110 | 15.1 | 130 | 48 | 349 | 43.6 |
| Urban | 173 | 36 | 3155 | 30.8 | 39 | 46.7 | 139 | 19.3 | 110 | 40.3 | 42 | 41.5 |
| Education Respondent | | |  |  |  |  |  |  |  |  |  |  |
| upto 10 years | 279 | 27 | 9106 | 16.9 | 11 | 49 | 134 | 17.6 | 172 | 43.5 | 21 | 53.8 |
| More than 10 years | 224 | 39.4 | 9282 | 26.7 | 242 | 55.2 | 115 | 18.2 | 68 | 49.3 | 370 | 42.8 |
| Desire for children | |  |  |  |  |  |  |  |  |  |  |  |
| Want soon | 461 | 33 | 16116 | 22.8 | 238 | 56 | 244 | 18.2 | 222 | 44.8 | 350 | 44.9 |
| Want no more/Others | 42 | 24.7 | 2272 | 17.3 | 15 | 33.5 | 5 | 0 | 18 | 58 | 41 | 30.7 |
| Wealth |  |  |  |  |  |  |  |  |  |  |  |  |
| Bottom 40% | 157 | 23.4 | 8649 | 15.1 | 116 | 61.7 | 122 | 11.1 | 101 | 34.6 | 179 | 40.8 |
| Top 60% | 346 | 36.5 | 9739 | 27.7 | 137 | 52 | 127 | 22.5 | 139 | 52.8 | 212 | 45.4 |
| Household size | |  |  |  |  |  |  |  |  |  |  |  |
| <=4 | 202 | 34.5 | 7183 | 25.1 | 56 | 68.6 | 111 | 18.9 | 50 | 51.5 | 210 | 43.8 |
| >4 | 301 | 30.8 | 11205 | 20.3 | 197 | 52.1 | 138 | 17 | 190 | 43.3 | 181 | 42.8 |
| Woman age | |  |  |  |  |  |  |  |  |  |  |  |
| 20-24 years | 175 | 39 | 11868 | 26.8 | 225 | 54.1 | 121 | 20.4 | 150 | 47.1 | 297 | 45.2 |
| 15-19 years | 328 | 28.9 | 6520 | 14.4 | 28 | 66.4 | 128 | 15.3 | 90 | 41.3 | 94 | 37.5 |
| Overall | 503 | 32.4 | 18388 | 22.2 | 253 | 55.1 | 249 | 17.9 | 240 | 45.3 | 391 | 43.3 |

Table S4: Prevalence of height less than 145 cm among married nulliparous adolescents and young women, South Asia

|  | Bangladesh DHS 2022 | | India DHS 2021 | | Maldives DHS 2017 | | Nepal DHS 2022 | | Pakistan DHS 2018 | | Sri Lanka DHS 2016 | |
| --- | --- | --- | --- | --- | --- | --- | --- | --- | --- | --- | --- | --- |
|  | N | % | N | % | N | % | N | % | N | % | N | % |
| Sector |  |  |  |  |  |  |  |  |  |  |  |  |
| Rural | 330 | 6.8 | 15233 | 12.7 | 214 | 4.9 | 110 | 7.3 | 130 | 5.3 | 349 | 5.7 |
| Urban | 173 | 2.8 | 3155 | 10.6 | 39 | 0 | 139 | 10.4 | 110 | 9.1 | 42 | 3.9 |
| Education Respondent | | |  |  |  |  |  |  |  |  |  |  |
| upto 10 years | 279 | 6.5 | 9106 | 16.1 | 11 | 0 | 134 | 10.9 | 172 | 8.7 | 21 | 16.1 |
| More than 10 years | 224 | 4.6 | 9282 | 8.9 | 242 | 2.6 | 115 | 7.6 | 68 | 1.9 | 370 | 5 |
| Desire for children | |  |  |  |  |  |  |  |  |  |  |  |
| Want soon | 461 | 5.8 | 16116 | 12.1 | 238 | 2.7 | 244 | 8.9 | 222 | 6.7 | 350 | 5.5 |
| Want no more/Others | 42 | 4.5 | 2272 | 13.5 | 15 | 0 | 5 | 34.9 | 18 | 3.5 | 41 | 5.5 |
| Wealth |  |  |  |  |  |  |  |  |  |  |  |  |
| Bottom 40% | 157 | 8 | 8649 | 17.1 | 116 | 6 | 122 | 11.9 | 101 | 7.8 | 179 | 6.2 |
| Top 60% | 346 | 4.6 | 9739 | 8.4 | 137 | 1 | 127 | 7.6 | 139 | 5.8 | 212 | 5 |
| Household size | |  |  |  |  |  |  |  |  |  |  |  |
| <=4 | 202 | 5.4 | 7183 | 13.2 | 56 | 3.8 | 111 | 5.4 | 50 | 4.7 | 210 | 5.8 |
| >4 | 301 | 5.8 | 11205 | 11.7 | 197 | 2.3 | 138 | 12.7 | 190 | 7.2 | 181 | 5.2 |
| Woman age | |  |  |  |  |  |  |  |  |  |  |  |
| 20-24 years | 175 | 6.1 | 11868 | 10.7 | 225 | 2.1 | 121 | 12.1 | 150 | 4.2 | 297 | 5.2 |
| 15-19 years | 328 | 5.5 | 6520 | 14.9 | 28 | 7.6 | 128 | 6.5 | 90 | 11.8 | 94 | 6.4 |
| Overall | 503 | 5.7 | 18388 | 12.3 | 253 | 2.5 | 249 | 9.4 | 240 | 6.6 | 391 | 5.5 |

Table S5: Country wise prevalence and burden of thinness, overweight and short stature among married nulliparous adolescents and young women, South Asia

|  | Bangladesh  DHS  2022 | India  DHS  2021 | Maldives  DHS 2017 | Nepal DHS  2016 | Pakistan DHS  2018 | Sri Lanka DHS 2016 |
| --- | --- | --- | --- | --- | --- | --- |
| Women who are 15-49 years (includes never married)^1^ | 38128 | 749344 | 9328 | 14845 | 25426 | 26767 |
| Nulliparous married women who are 15-24 years^1^ | 503 | 18388 | 253 | 249 | 240 | 391 |
| Nulliparous married women who are 15-19 years^1^ | 328 | 6520 | 28 | 128 | 90 | 94 |
| Nulliparous married women who are 20-24 years^1^ | 175 | 11868 | 225 | 121 | 150 | 297 |
| Nulliparous married women 15-24 years as a share of 15-49 years^1^ | 0.013 | 0.025 | 0.027 | 0.017 | 0.009 | 0.015 |
| Nulliparous married women 15-19 years as a share of 15-24 years^1^ | 0.65 | 0.35 | 0.11 | 0.51 | 0.38 | 0.24 |
| Nulliparous married women 20-24 years as a share of 15-24 years^1^ | 0.35 | 0.65 | 0.89 | 0.49 | 0.63 | 0.76 |
| Women who are 15-49 years^2^ | 48003000 | 368336618 | 119988 | 8930435 | 53366324 | 5453498 |
| Nulliparous married women who are 15-24 years | 633275 | 9038537 | 3254 | 149793 | 503733 | 79662 |
| Nulliparous married women who are 15-19 years | 412951 | 3204876 | 360 | 77002 | 188900 | 19152 |
| Nulliparous married women who are 20-24 years | 220324 | 5833661 | 2894 | 72791 | 314833 | 60511 |
| Prevalence |  |  |  |  |  |  |
| Severe thin | 2.4 | 4.3 | 7.1 | 1.8 | 3.2 | 4.6 |
| Thin | 18.2 | 25.9 | 15.9 | 22.4 | 14.6 | 18.4 |
| Overweight | 32.4 | 22.2 | 55.1 | 17.9 | 45.3 | 43.3 |
| Low stature | 5.7 | 12.3 | 2.5 | 9.4 | 6.6 | 5.5 |
| Burden 15-24 |  |  |  |  |  |  |
| Severe thin | 15199 | 388657 | 231 | 2696 | 16119 | 3664 |
| Thin | 115256 | 2340981 | 517 | 33554 | 73545 | 14658 |
| Overweight | 205181 | 2006555 | 1793 | 26813 | 228191 | 34494 |
| Low stature | 36097 | 1111740 | 81 | 14081 | 33246 | 4381 |

Source: ^1^Based on DHS and ^2^World Population Prospects

### Table S6: Prevalence of severe thinness (BMI < 16 kg/m2) among married nulliparous adolescents and young women, South Asia

|  |  | Adolescents and Married Women | |
| --- | --- | --- | --- |
|  |  | N | BMI < 16 kg/m2 (%) |
| Bangladesh | DHS 2018 | 1048 | 2.2 |
|  | DHS 2022 | 503 | 2.4 |
| India | DHS 2016 | 22515 | 4.7 |
|  | DHS 2021 | 18388 | 4.3 |
| Maldives | DHS 2009 | 346 | 4.2 |
|  | DHS 2017 | 253 | 7.1 |
| Nepal | DHS 2016 | 286 | 1.3 |
|  | DHS 2022 | 249 | 1.8 |
| Pakistan | DHS 2013 | 215 | 1.2 |
|  | DHS 2018 | 240 | 3.2 |
| Sri Lanka | DHS 2016 | 391 | 4.6 |
| Poole | Earlier | 24410 | 4 |
|  | Latest | 20024 | 4.1 |

Table S7: Prevalence of anemia among married nulliparous adolescents and young women, South Asia

|  |  | Adolescents and Married Women | |
| --- | --- | --- | --- |
|  |  | N | Anemia (%) |
| India | DHS 2016 | 22515 | 52.8 |
|  | DHS 2021 | 18388 | 58.3 |
| Maldives | DHS 2009 | NA | NA |
|  | DHS 2017 | 243 | 59.6 |
| Nepal | DHS 2016 | 286 | 39.4 |
|  | DHS 2022 | 249 | 38.2 |
